# Exploring the capture and desorption of CO_2_ on graphene oxide foams supported by computational calculations

**DOI:** 10.1038/s41598-023-41683-4

**Published:** 2023-09-02

**Authors:** Bryan E. Arango Hoyos, H. Franco Osorio, E. K. Valencia Gómez, J. Guerrero Sánchez, A. P. Del Canto Palominos, Felipe A. Larrain, J. J. Prías Barragán

**Affiliations:** 1 Energy Engineering, Faculty of Engineering and Sciences, Universidad Adolfo Ibáñez, 7941169 Santiago, Chile; 2 Electronic Instrumentation Technology Program, Faculty of Basic Science and Technology, Universidad del Quindío, 630001 Armenia, Colombia; 3 Doctoral Program in Physical Sciences, Interdisciplinary Institute of Sciences, Universidad del Quindío, 630004 Armenia, Colombia; 4 Virtual Materials Modeling Laboratory (LVMM), Center for Nanoscience and Nanotechnology, Universidad Nacional Autónoma de México, Ensenada, 22860 Mexico

**Keywords:** Environmental chemistry, Environmental sciences, Chemistry, Environmental chemistry, Theoretical chemistry, Theory and computation

## Abstract

In the last decade, the highest levels of greenhouse gases (GHG) in the atmosphere have been recorded, with carbon dioxide (CO_2_) being one of the GHGs that most concerns mankind due to the rate at which it is generated on the planet. Given its long time of permanence in the atmosphere (between 100 to 150 years); this has deployed research in the scientific field focused on the absorption and desorption of CO_2_ in the atmosphere. This work presents the study of CO_2_ adsorption employing materials based on graphene oxide (GO), such as GO foams with different oxidation percentages (3.00%, 5.25%, and 9.00%) in their structure, obtained via an environmentally friendly method. The characterization of CO_2_ adsorption was carried out in a closed system, within which were placed the GO foams and other CO_2_ adsorbent materials (zeolite and silica gel). Through a controlled chemical reaction, production of CO_2_ was conducted to obtain CO_2_ concentration curves inside the system and calculate from these the efficiency, obtained between 86.28 and 92.20%, yield between 60.10 and 99.50%, and effectiveness of CO_2_ adsorption of the materials under study. The results obtained suggest that GO foams are a promising material for carbon capture and the future development of a new clean technology, given their highest CO_2_ adsorption efficiency and yield.

## Introduction

**Figure 1:**
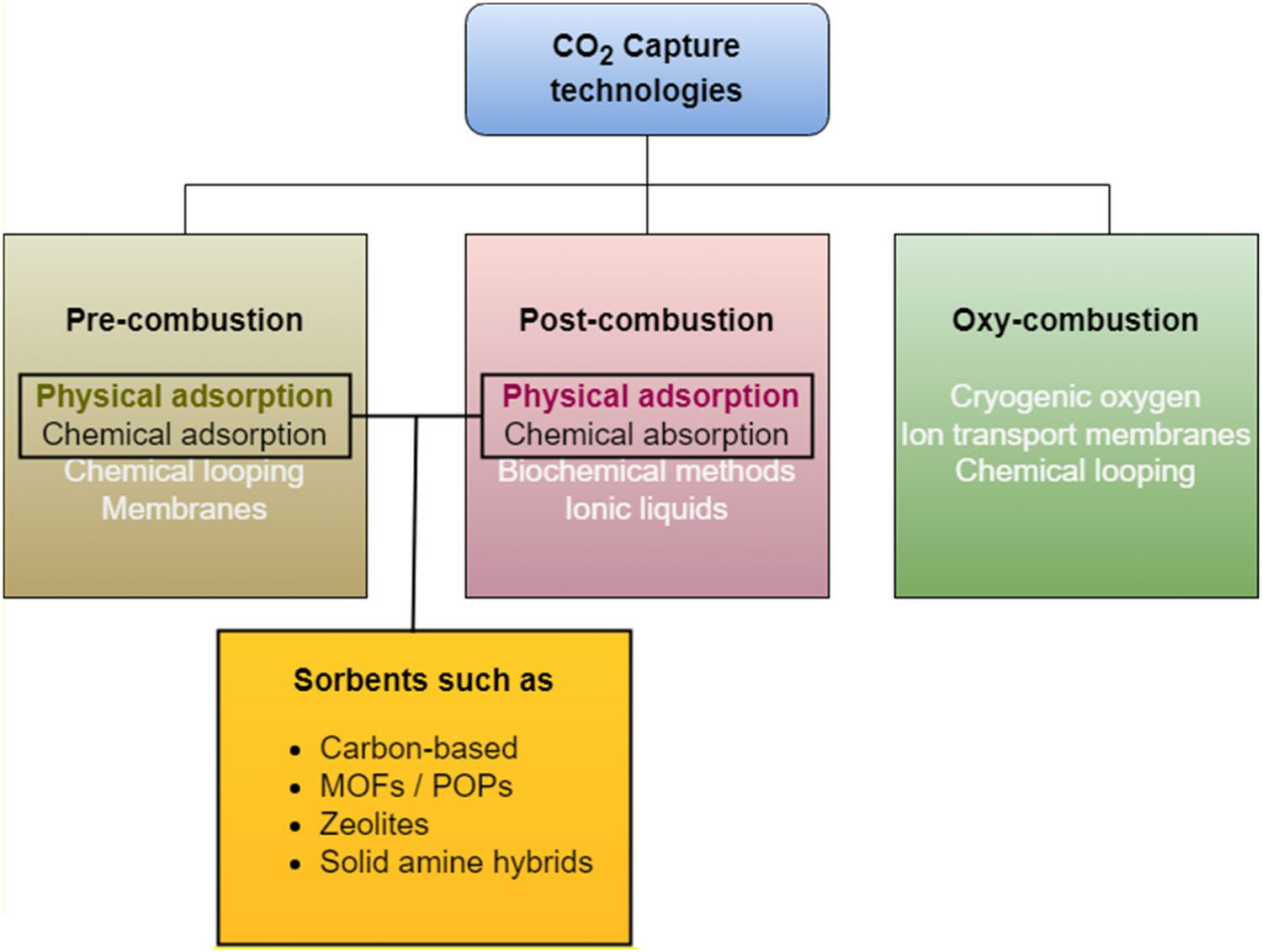
CCS technologies for industrial waste, listing sorbents which can be applied to capture CO_2_ from air.

Carbon capture and storage (CCS) is becoming a hot topic as the urgency to contain climate change grows^1^. Solutions to capture CO_2_ from highly concentrated sources (that is, CO_2_ concentrated at a 10% level or more) exist and have been around for decades. These solutions have been applied extensively to exhaust vents in industrial processes and may be categorized depending upon the stage in which they are incorporated: pre-combustion, post-combustion, or oxy combustion^2^ (see Fig. 1). Regardless of the technology, these systems operate following two main steps: capture and release of CO_2_. Put simply, CO_2_-containing gas is blown into a contactor that contains material with the ability to capture CO_2_. Then, some of the CO_2_ present in the gas stream is captured. Next, the CO_2_ is released by applying energy, vacuum, moisture, or a combination of them, to move it into further sequestration or utilization. This way, the CO_2_-capturing material is taken back to its original state, (or “regenerated”), so the cycle can be restarted.

While capturing CO_2_ from industrial processes is relatively mature, capturing the same molecule from air is still considered an emerging technology. It turns out that capturing CO_2_ from highly diluted sources (in air, CO_2_ oscillates approximately between 410 and 420 ppm, which represents a level of concentration of 0.041%) is a completely different problem, were point-source CO_2_-capture technologies are not directly applicable. This is why the scientific community has been studying a wide range of materials which could serve as sorbents (either physisorbents or chemisorbents), depending upon their stability, selectivity to CO_2_, surface area, porosity capture capacity, and other properties^3^. This work focuses on adsorption, that is, solid materials which can capture CO_2_ from air.

Despite the recent progress, data on the stability and longevity of adsorbents are scarcely available in the literature, as show in Table 1. On the one hand, there is no single experimental method or tools to characterize adsorbent stability. While some researchers use thermal gravimetry and test a pelletized form of a composite that includes the adsorbent, others conduct sorption and desorption cycling in a fixed bed, in what is referred to as the “capture experiment”. Among them, some build structured contactors instead of testing pelletized composite. As a result, summarizing the state of the art of CO_2_-adsorbing materials and their properties may involve comparing data which is not strictly comparable. Out of completeness, Table 1 includes a revision of some of the most studied adsorbing materials and their properties.

**Table 1:** Stability data from pelletized composites and structured adsorbents adsorption/desorption cycles.

Materials	Method	Sample	Cycle time (min)	Stability evaluation (cycles)	Average capacity (mmolCO_2_/g adsorbent)	Ref.
PEI/Ti-SBA-15 (4.3)	TVSA	Structured adsorbent	369	4	1.25	^4^
SI-AEATPMS	TVA	Pelletized composite	255	40	0.05	^5^
PEI/silica	TGA	Pelletized composite	465	4	2.26	^6^
HAS-5.4	TGA	Pelletized composite	244.5	4	2.13	^7^
PL-0.75	TGA	Pelletized composite	22.5	3	0.50	^8^
TRI-PE-MCM-41 (dry)	TGA	Pelletized composite	195	4	6.25	^9^
FS-LPEI (5000)	TSA	Pelletized composite	39	100–200	0.01	^10^
PPI/SBA-15	TVSA	Pelletized composite	70	50	0.26	^11^
TEPA-PO-1-2/50S	TSA	Pelletized composite	210	15	0.04	^12^
en-Mg2(dobpdc)	TGA	Pelletized composite	397.5	5	1.20	^13^
Cr-MIL-101-SO3H-TAEA	TSA/TVSA	Pelletized composite	22.5	15	0.17	^14^
Amine PEI alumina 10%	TGA	Pelletized composite	131	1	0.24	^15^
Mg2 (dobdc) with EDA	–	Structured adsorbent	–	1	N/I	^16^
bPEI/SBA-15	TGA	Pelletized composite	10	20	1.0	^17^
PEI/SBA-15	TGA	Pelletized composite	30	4	0.2	^18^
PPI/SBA-15	TGA	Pelletized composite	6000	50	1.75	^19^
polyHIPE	–	Structured adsorbent	300	5	0.7	^20^
MC-1.5-60	TGA	Pelletized composite	100	10	4.4	^21^
amine-modified	–	porous adsorbents	–	10	0.5	^22^

*TGA* thermal gravimetry analysis, *TSA* temperature swing adsorption, *TVSA* temperature vacuum adsorption.

Additionally, other porous materials such as zeolite-based molecular sieves, activated carbons (ACs), and carbon nanotubes (CNTs) have attracted attention from researchers for gas adsorption. Activated carbons generally provide greater additional capacity at pressures above atmospheric pressure, compared to zeolites. In addition, ACs are often preferred over zeolites due to their relatively moderate gas adsorption strength, which facilitates desorption^23–26^. Furthermore, Zhang and collaborators^27^ have studied the microporous *n*-doped carbon adsorbent, obtained using polyaniline as a precursor, denoting that pore size and quantity play a critical role in the capture of CO_2_ in this type of material. Other studies employing wood sawdust and transforming it into biochar by a pyrolysis method have been carried out. Remarkably, it was found that the processing temperature impacts not only the yield but also the CO_2_ adsorption capacity of the material^28^.This is why it would be interesting to examine other adsorbents derived from vegetation waste, like graphene oxide.

Graphene is an increasingly important material and its storage capacity for different gasses has been suggested in theoretical studies; CO_2_ adsorption capacity is demonstrated at very low temperatures (195 K), which does not have much practical implication^29^. Therefore, it is necessary to investigate the CO_2_ adsorption capacity of graphene at room temperature and moderate pressure for the practical application of graphene in carbon capture and storage (CCS) technology^30^. In this work, GO synthesized by the Double Thermal Decomposition (DTD) method^31^ at different temperatures is used in the interdisciplinary Institute of Sciences at Universidad del Quindío in cooperation with Universidad Adolfo Ibáñez.

Graphene has shown intensive and promising applications in electronic devices^32^, batteries^33^, and composites^34,35^. Researchers have developed many methods to prepare this promising new nanomaterial, such as mechanical exfoliation, chemical vapor deposition (CVD)^36^, transfer printing^37^, epitaxial growth^38^, organic synthesis^39^, and oxidation-dispersion-reduction. Among these methods, the chemical reduction of GO sheets can produce graphene in large quantities, employing graphite as raw material. Because graphite is cheap and readily available, this chemical approach is probably the least expensive, most effective method for the large-scale production of graphene^40^.

Evidence, to date, has determined that graphene is a *sp*^2^-bonded planar carbon material. Due to its great potential in electronic applications, it has attracted much attention since it was first isolated in 2004. Driven by a fundamental interest and potential applications, but also as an example of chemical functionalization, graphene oxidation has been intensively studied^41–43^. However, due to the amorphous nature of GO generated by the chemical manufacturing method, understanding the atomic structure and its effects on the oxidation process remains a major challenge^44–53^.

Some authors report working with GO using hybrid materials and postulate them as potential materials for CO_2_ capture^31,54–58^. Other authors impregnated materials, like Zeolite and Silica gel, with amines. Amine-functionalized porous materials outperform all others in terms of CO_2_ adsorption capacity and regeneration efficiency^59,60^. Moreover, thermodynamic changes in systems where the GO is found can help us to look for desorption points, whether at high or low temperatures^61–69^.

Thus, studies of CO_2_ adsorption in GO structures in foams (GO-Foams) obtained through a carbonization process (873.15–1053.15 K) of organic waste material were carried out and additional tests on two materials derived from coffee as non-adsorbing reference materials can be found in Supplementary Information. In addition, adsorption calculations for a CO_2_ molecule on the surface of graphene and GO were also estimated. Therefore, the performance comparison between non-carbon (Zeolite and silica gel) material and the GO-Foams derived from vegetation waste is reported here. Furthermore, this work presents a functional application for this material in highly contaminated urban environments.

## Materials and methods

### Characterization method

**Figure 2:**
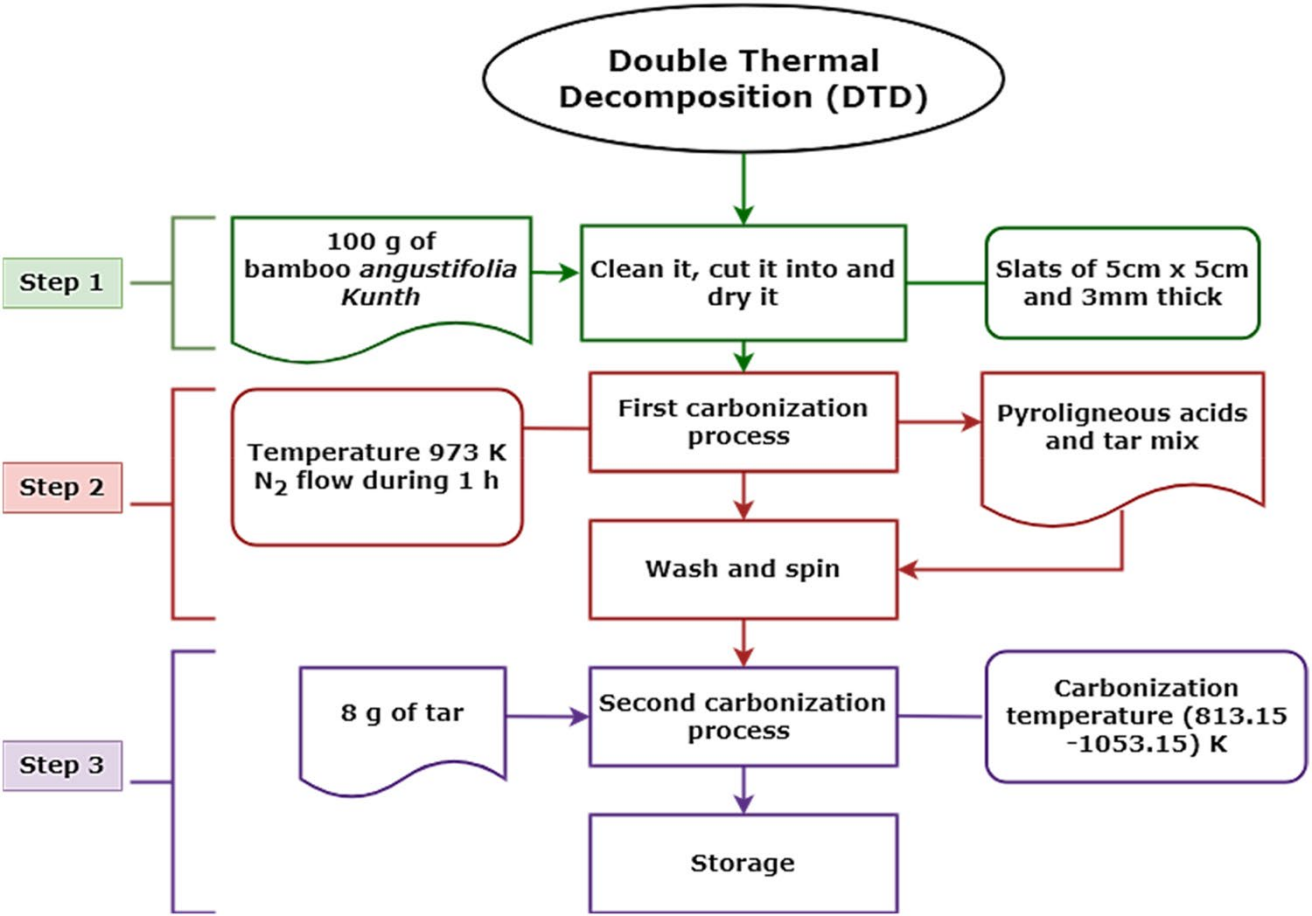
Flowchart for the DTD method to obtain the GO foams used in this research.

Synthesis of GO foam was carried out by employing an efficient and environmentally friendly method, so-called the double thermal decomposition method (DTD), as reported^70^ and presented in a flowchart in Fig. 2. The method consists of treating a waste product of commercial bamboo—*Guadua angustifolia* Kunth—at different carbonization temperatures. In step 1, biomass from bamboo gets passivated, cleaned, and cut to move forward to step 2, where the first pyrolysis is carried out. The tar resulting from this step is taken to a second pyrolysis in which the GO foam is obtained, as noted in Fig. 4a–c. The authors confirm that all methods in experimental research and field studies on plants, as a waste product of the commercial bamboo-Guadua, were performed in accordance with the relevant regulations. Furthermore, the oxidation degree of graphene oxide was previously correlated to the carbonization temperature through XPS analyses, which were reported before^53^. The material was also characterized using TEM, XRD and Raman spectroscopy, as shown in Fig. 4.

Here, GO foams were synthesized at 873 K (9.00% oxidation), 973 K (5.25% oxidation), and 1053 K (3.00% oxidation), which oxidation rate was determined via XPS analyses, as reported before^71^. Table 2 shows the three oxidation rates of GO with their respective formation temperatures, the time elapsed, superficial area and porosity. The authors confirm that all methods in experimental research and field studies on plants were performed adhering to relevant regulations^70,72,73^.

**Table 2:** Types of graphene oxide used for the experiments herein.

Oxidation rate	T_CA_* (K)	Time (h)	Superficial area (m^2^/g)	Porosity (µm)
GO-9.00%	873.15	1	570.9	21.8
GO-5.25%	973.15	1	471.2	22.2
GO-3.00%	1053.15	1	403.9	23.1

*T_*CA*_ Carbonization temperature.

### CO_2_ adsorption characterization

The characterization of CO_2_ adsorption of GO foams was carried out in an isolated CO_2_ measurement system based on the use of the MHZ-19B reference CO_2_ sensor in parts per million (ppm)^74^, which presents an optical measurement mechanism, allowing accurate measurements to be obtained in a wide range, from 0 to 5000 ppm ± 50 ppm. For this, a reaction for CO_2_ generation was introduced at the bottom of a closed system; this reaction is based on the reaction given by Eq. (1).

1 $${\text{NaHCO}}_{{{3}({\text{solid}})}} + {\text{ CH}}_{{3}} {\text{COOH}}_{{({\text{liquid}})}} \to {\text{CH}}_{{3}} {\text{COONa}}_{{({\text{liquid}})}} + {\text{ H}}_{{2}} {\text{O}}_{{({\text{liquid}})}} + {\text{ CO}}_{{{2}({\text{gas}})}}$$

**Figure 3:**
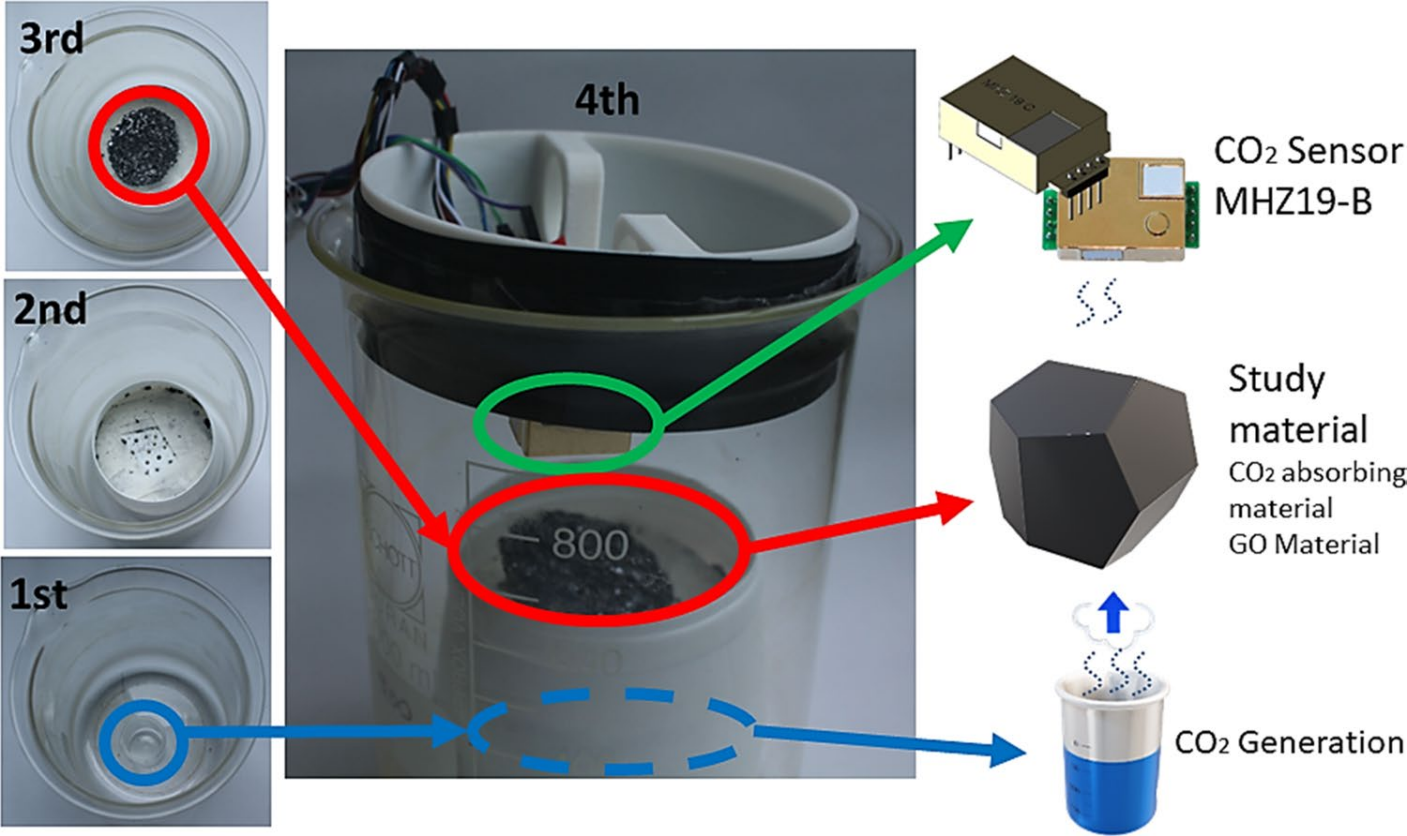
CO_2_ adsorption characterization system (GO–Foam–CO_2_) for GO foams developed herein.

To guarantee controlled CO_2_ production within the system, two compounds were used: acetic acid and sodium bicarbonate, which give as product three other compounds: sodium acetate, water (H_2_O), and carbon dioxide (CO_2_), making it an efficient and low-cost CO_2_ production. An MH-Z19B CO_2_ sensor is located above of the GO foam to ensure better reading of the CO_2_ adsorption (Fig. 3). In the first part of Fig. 3, the CO_2_ source (NaHCO_3_ (solid) + CH_3_COOH (liquid) reaction) is located in the lower part of the experiment, and in the second part of Fig. 3, our sample holder is located in said CO_2_ source, followed by the third part; for this, the material under study is located on the sample holder and, thus, said material is located in our gas source. Finally, as a fourth part, the system is sealed with the upper cover (which has the sensors) that will prevent the gas from leaking into the system.

### Computational details

The VASP software was used to calculate the molecules^75^. To perform the geometric and energetic calculations, the GGA functional, PBE^76^ was used because this functional is widely employed to predict various properties of molecules and non-bond interactions^77^. Based on the Lerf–Klinowski model^78,79^ and the structure presented by Prías-Barragán et al.^72^ a single CO_2_ molecule and two structures of isolated armchair graphene flakes were modeled; the first one is graphene with hydrogen passivated edges (${C}_{100}{H}_{26}$), and a GO structure with 9.00% oxide coverage (${C}_{100}{H}_{34}{O}_{9}$), given that the GO employed in the experimental case is in the lower oxidation regimen^70^. After the first relaxation of every structure, the CO_2_ molecule was placed at a certain distance from the graphene and GO surface and the process was repeated. To obtain the adsorption energies, Eq. (2) was employed,

2 $${E}_{ads}={E}_{system}-{E}_{graphene}-{E}_{C{O}_{2}}$$

where ${E}_{system}$ corresponds to the energy of the graphene or GO sheet with a CO_2_ molecule adsorbed, and ${E}_{graphene}$ and ${E}_{C{O}_{2}}$ correspond to the energy associated with the isolated graphene and CO_2_ molecule, respectively.

The non-covalent interactions (NCI) and molecular electrostatic potential (MEP) were calculated to analyze theoretically the adsorption of the CO_2_ molecule on graphene and GO structures. The charge transfer was examined by analyzing Bader charges, obtained through the critic2 software^80,81^, before and after adsorption. Most of the calculations were performed in the cluster from the Virtual Materials Modeling Laboratory (VMML) group, at the Center for Nanoscience and Nanotechnology, in the “Miztli” supercomputer, with a processing capacity of 228 TFlop/s, which has 8,344 processing cores, 16 NVIDIA m2090 cards, a total RAM of 45,000 GB and a 750 TB mass storage system, property of UNAM.

## Results and discussions

**Figure 4:**
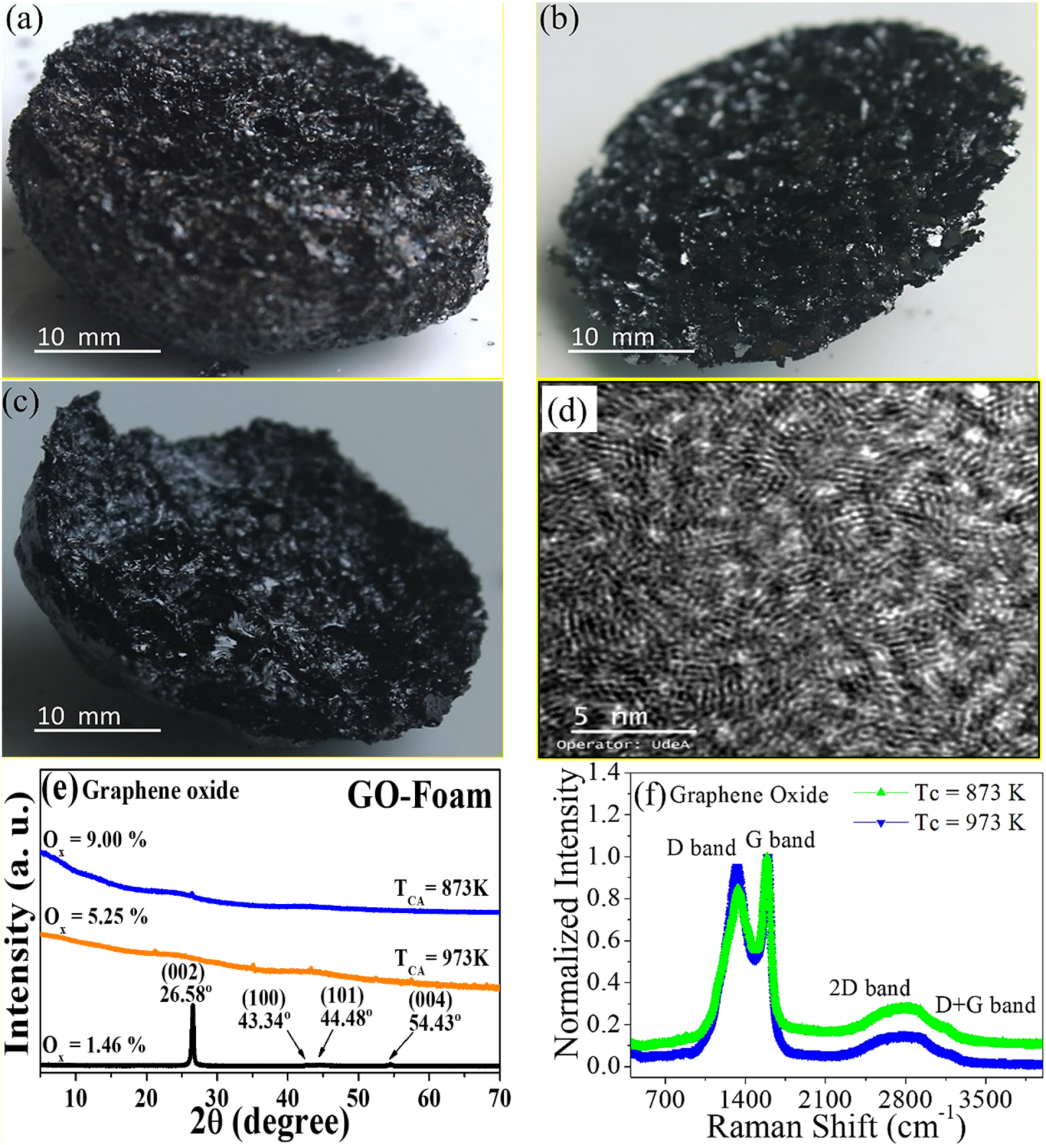
GO foams obtained employing the DTD and characterization methods, (**a**) 873.15 K (GO 9.00%), (**b**) 973.15 K (GO 5.25%), (**c**) 1053.15 K (GO 3.00%), (**d**) GO–TEM, (**e**) GO–XRD patterns and (**f**) GO–Raman at 873 and 973 K.

Figure 4a–c shows photographs of graphene oxide foams at different oxidation rates. In Fig. 4d the transmission electron microscopy of the GO is observed. These graphene foams have a close porosity as seen in Table 2, this allowing the entry and exit of CO_2_ gas. The Fig. 4e presents the consolidated XRD patterns of GO–Foam samples synthesized at different T_CA_, observing in the GO–Foam samples the characteristic peaks of hexagonal Graphite in the (002), (100), (101), and (004) directions, showing that it is a polycrystalline material. Figure 4f illustrates normalized Raman spectra of GO-Foam samples, presenting the characteristic peaks G-band peak around 1560 cm^−1^ associate to graphene structure and D-band peak around 1350 cm^−1^ attribute to the disorder-induced phonon mode; The wide 2D and D + G bands around the 2800 cm^−1^ value suggest the presence of multiple graphene layers with edges, defects, and sp^2^ regions, which are prevalent features of the GO − Foams synthetized, as previously reported^70,82,83^.

### CO_2_ generation

To calibrate and fine-tune the CO_2_ sensors, CO_2_ was produced from a reaction of NaHCO_3_ (as solid, sodium bicarbonate) plus CH_3_COOH_(aqueous)_ (acetic acid), yielding CH_3_COONa _(aqueous)_ (sodium acetate), plus H_2_O_(liquid)_ (water), plus CO_2(gas)_ (carbon dioxide).

**Figure 5:**
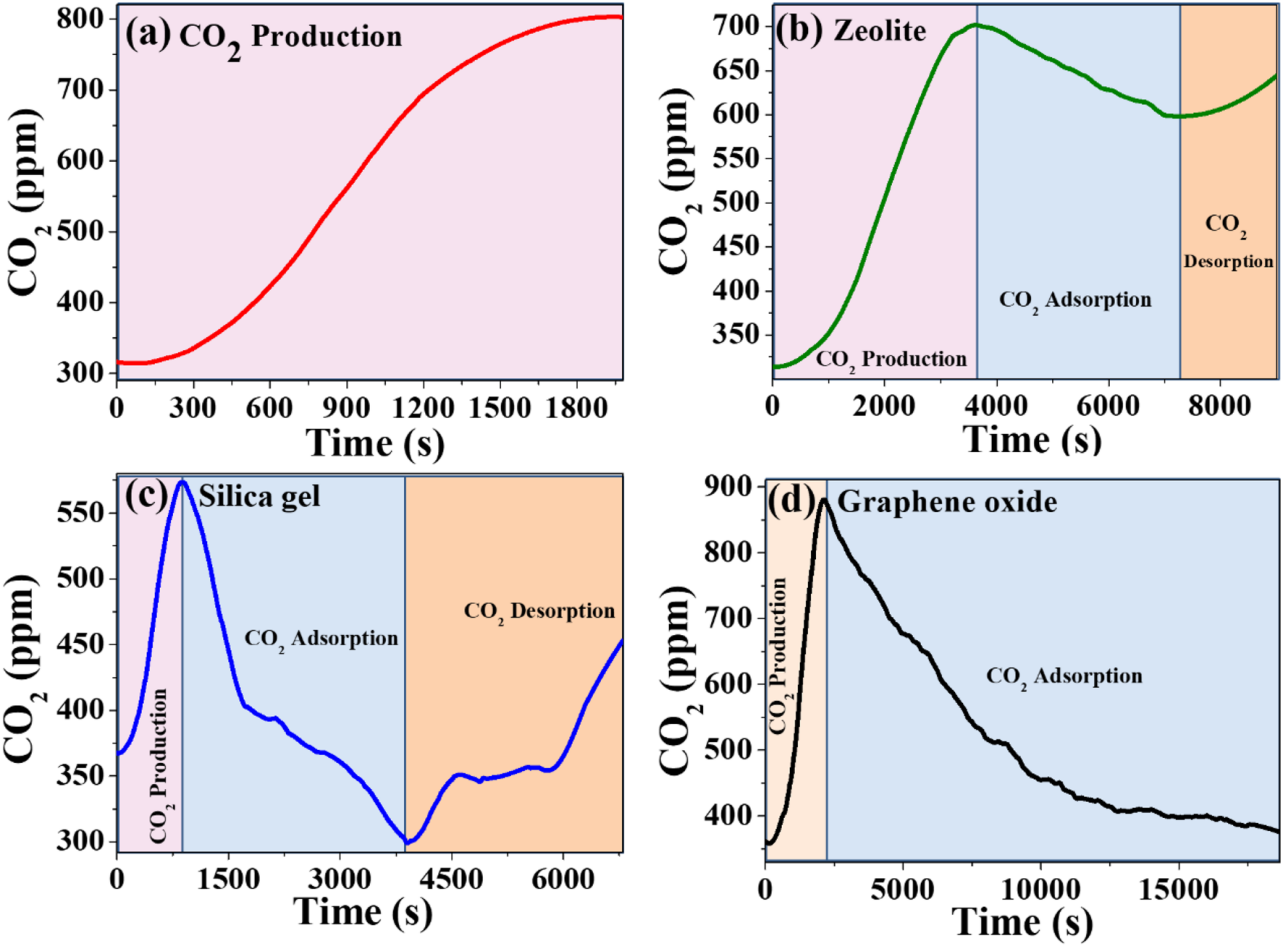
(**a**) CO_2_ generation characterization, CO_2_ adsorption in (**b**) Zeolite, (**c**) Silica gel, and (**d**) CO_2_ adsorption using GO-9.00% at 294.15 K.

Initially, a measurement of CO_2_ production was performed inside the system from the reaction of 1.5 mg of NaHCO_3 (solid)_ (sodium bicarbonate) plus 0.5 ml of CH_3_COOH _(aqueous)_ (acetic acid), yielding CH_3_COONa _(aqueous)_ (acetate of sodium), plus, H_2_O _(liquid)_ (water), plus, CO_2 (gaseous)_ (carbon dioxide), as products. Figure 5a identifies the CO_2_ production obtained, where the concentration of this gas increases from 325 to approximately 800 ppm.

### CO_2_ adsorption on zeolite, silica gel, and graphene oxide foam

The graph in Fig. 5b, where zeolite was used as an absorbent material, shows CO_2_ concentration vs. time in seconds, starting with a minimum CO_2_ concentration of 280 ppm; CO_2_ production was observed with an approximate maximum of 700 ppm at 3,500 s after starting the CO_2_ production reaction. After this time, absorption of the zeolite is evident with adsorption reaching 575 ppm at 7000 s and, thereafter, it is observed that it does not contain CO_2_ within for a long time, again showing CO_2_ release, increasing to 650 ppm. In Fig. 5c, in the presence of silica gel as adsorbent material, the graph shows CO_2_ concentration vs. time in seconds, starting with a minimum CO_2_ concentration of 370 ppm and CO_2_ production with an approximate maximum observed, from 600 ppm at 1200 s after starting the CO_2_ production reaction. After this time, the absorption of the silica gel is evident with adsorption reaching 300 ppm at 4000 s and later it is observed that it does not contain CO_2_ inside for a long time, again showing CO_2_ release, increasing to 450 ppm. This indicates its low retention capacity inside its structure. Figure 5d, presents the evolution of the CO_2_ production in GO-9.00% foam at 294.15 K (room temperature) in a closed system shown in Fig. 3. After approximately 2500 s, a clear slow absorption of CO_2_ in the system is noted, thus, revealing a slow decrease in CO_2_ gas, with slow adsorption over time until stable departure levels are reached. When comparing these three adsorbent materials, the superiority of the oxidized graphene foam is identified concerning zeolite and silica gel, given that they contain more CO_2_ gas and maintain it over time due to their high efficiency and performance.

### Temperature effects on the saturation of CO_2_ adsorbed on GO foam

**Figure 6:**
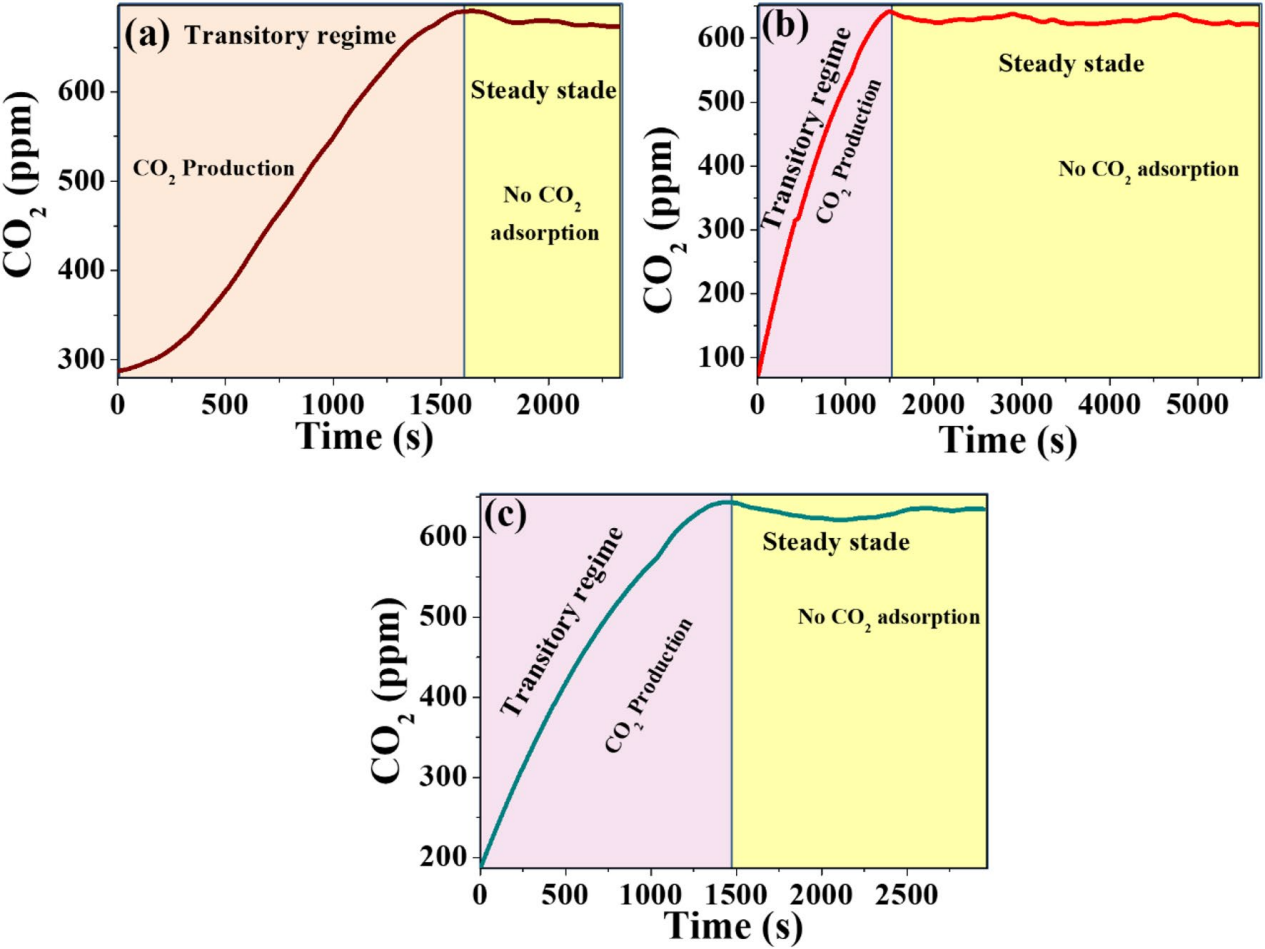
(**a**) GO-9.00% with a temperature of 423.15 K, (**b**) GO-9.00% with a temperature of 523.15 K, and (**c**) GO-9.00% with a temperature of 573.15 K.

In the experiment using GO-9.00% for CO_2_ adsorption, this gas was produced using 1.5 mg NaHCO_3_ (solid) (sodium bicarbonate) and 0.5 ml CH_3_COOH (aqueous) (acetic acid). The graphene oxide used in the experiment was heated to 423.15 K for 48 h and, subsequently, it was measured if it had already released CO_2_ from its interior, as seen in Fig. 6a. A constant trend of stability in CO_2_ production is determined, starting from 300 ppm within the system up to 700 ppm of production, this last value identified as constant, from 1500 s on; this is attributed to the fact that this CO_2_ gas was not released due to a stationary regime. CO_2_ Gas in the GO at 423.15 K cannot be retained on its walls, and therefore could not adsorb more CO_2_ gas; since it requires more energy to desorb and thus be able to be ready for a new adsorption. Therefore, this GO foam continued to heat up further. This also occurred with the other two temperatures explored before knowing the ideal desorption temperature of the GO foam using heating temperatures of 523.15 K, as seen in Fig. 6b, starting from 100 ppm and obtaining a maximum CO_2_ production of 650 ppm, remaining stable at this value. When heated to 573.15 K, it was again exposed to a CO_2_ reaction, where at 573.15 K (Fig. 6c) it is observed that it departs from a CO_2_ concentration of 200 ppm, reaching a maximum of 650 ppm and then remaining stable in a valley evidencing that there is no adsorption of the gas due to a stationary regime. It is important to note that the exact temperature dependence of CO_2_ adsorption on GO foams will depend on the specific properties of the foam, such as pore size, surface area, and functional groups. Therefore, experimental studies are needed to determine the temperature dependence of CO_2_ adsorption on a particular GO foam.

### Temperature influence on the re-adsorption of CO_2_ adsorbed on GO foam

**Figure 7:**
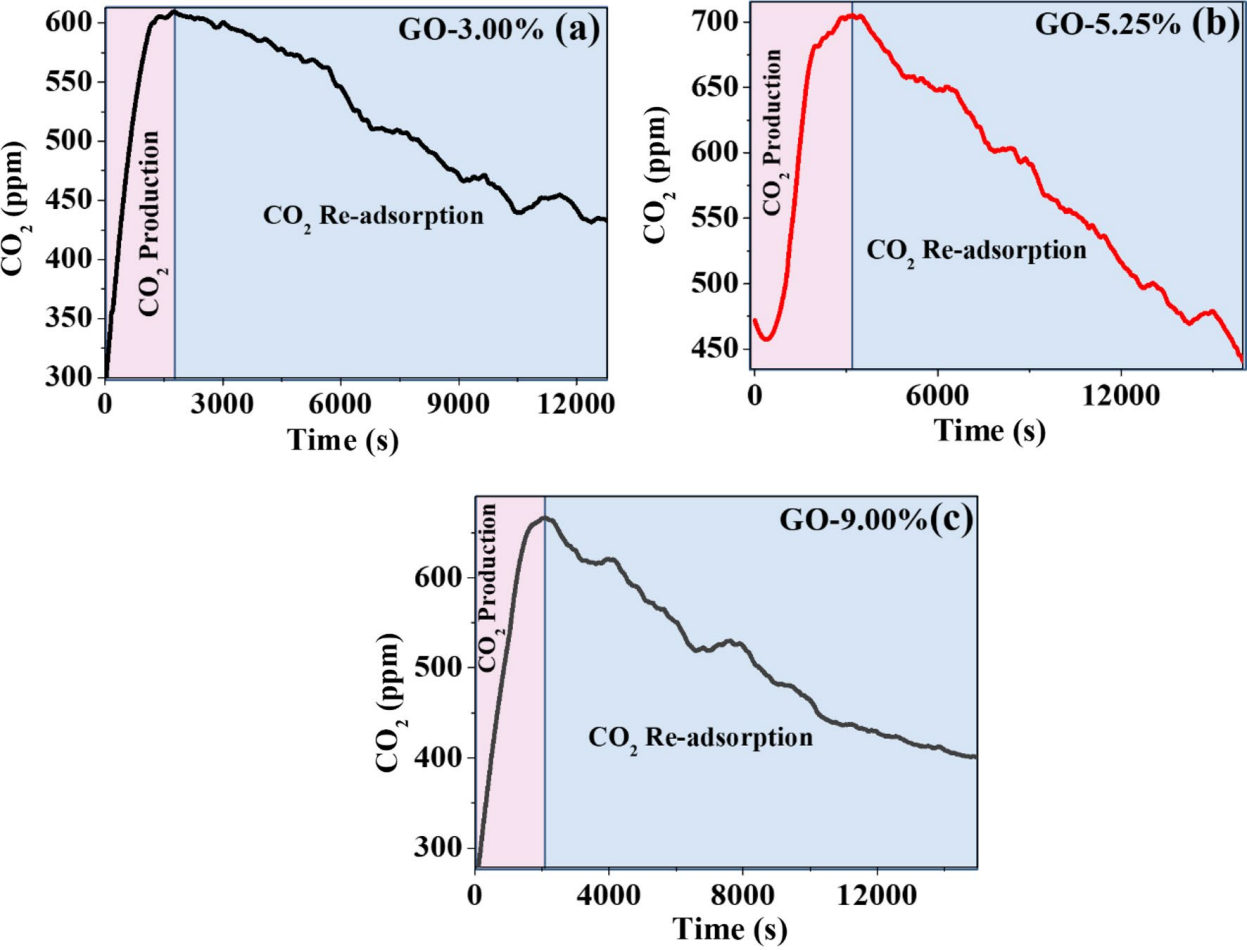
(**a**) GO-3.00% with re-adsorption temperature of 673.15 K, (**b**) GO-5.25% with re-adsorption temperature of 673.15 K, and (**c**) GO-9.00% with re-adsorption temperature of 673.15 K.

The GO-3.00% already saturated with CO_2_ was used, which was synthesized at 1053.15 K. The same graphene from the previous experiments was used, already saturated with CO_2_, placed in a muffle, and heated to 673.15 K for 5 h and 30 min. Desorption results were successful because the material recovered its adsorbent condition, as shown in Fig. 7a, going from a CO_2_ reduction from 600 to 420 ppm in 12,000 s, to again show its adsorption qualities. This results in a great quality of CO_2_ adsorption and desorption, called re-adsorption. Re-adsorption of CO_2_ on GO foams can be influenced by temperature in several ways; solubility of CO_2_ in a material decreases with increasing temperature. However, CO_2_ re-adsorption onto GO foam is a complex process involving multiple mechanisms, so the effect of temperature on re-adsorption may not be straightforward, as seen in this work. GO-5.25%, already saturated with CO_2_ was used, synthesized at 973.15 K. This already saturated graphene from the previous experiments was used, placed in a muffle, and heated to 673.15 K for 5 h and 30 min. The desorption results were successful because the material recovered its adsorbent condition, as shown in Fig. 7b, going from a CO_2_ reduction from 700 to 450 ppm in 16,000 s, to then also show its re-adsorption qualities, thus improving the results of GO-3.00%. Physisorption is a process in which CO_2_ gas molecules are held to a surface by weak van der Waals forces. The interaction of these forces increases as temperature decreases, thereby, lowering the temperature may increase the amount of CO_2_ that can be physiosorbed onto the GO foam or, conversely, if temperature is increased these forces are weakened, thus allowing the GO-Foam-CO_2_ to desorb. Another mechanism that can be influenced by temperature is chemisorption. Chemisorption is a chemical reaction between the adsorbate (CO_2_) and the adsorbent (GO foam), which can be exothermic or endothermic, depending on the specific reaction. Changes in temperature can affect the activation energy of the reaction and the energy required for the adsorption process, which—in turn—can affect the rate and extent of re-adsorption. GO-9.00% already saturated with CO_2_ was used, synthesized at 973.15 K. This already saturated graphene from the previous experiments was used, placed in a muffle, and heated to 673.15 K for 5 h and 30 min. The desorption results were successful because the material recovered its adsorbent condition, as shown in Fig. 7c, going from a CO_2_ reduction from 750 to 400 ppm in 15,000 s, to again begin to show its adsorption qualities; resulting in a great property of CO_2_ re-adsorption. It is evident that it did not improve the conditions of the results of the GO-5.25% but did improve those of the GO-3.00%. In summary, temperature can influence CO_2_ re-adsorption on GO foam through physisorption and chemisorption mechanisms. The specific effect of temperature will depend on the specific conditions and properties of the GOFs and the CO_2_ gas.

### Low temperatures

**Figure 8:**
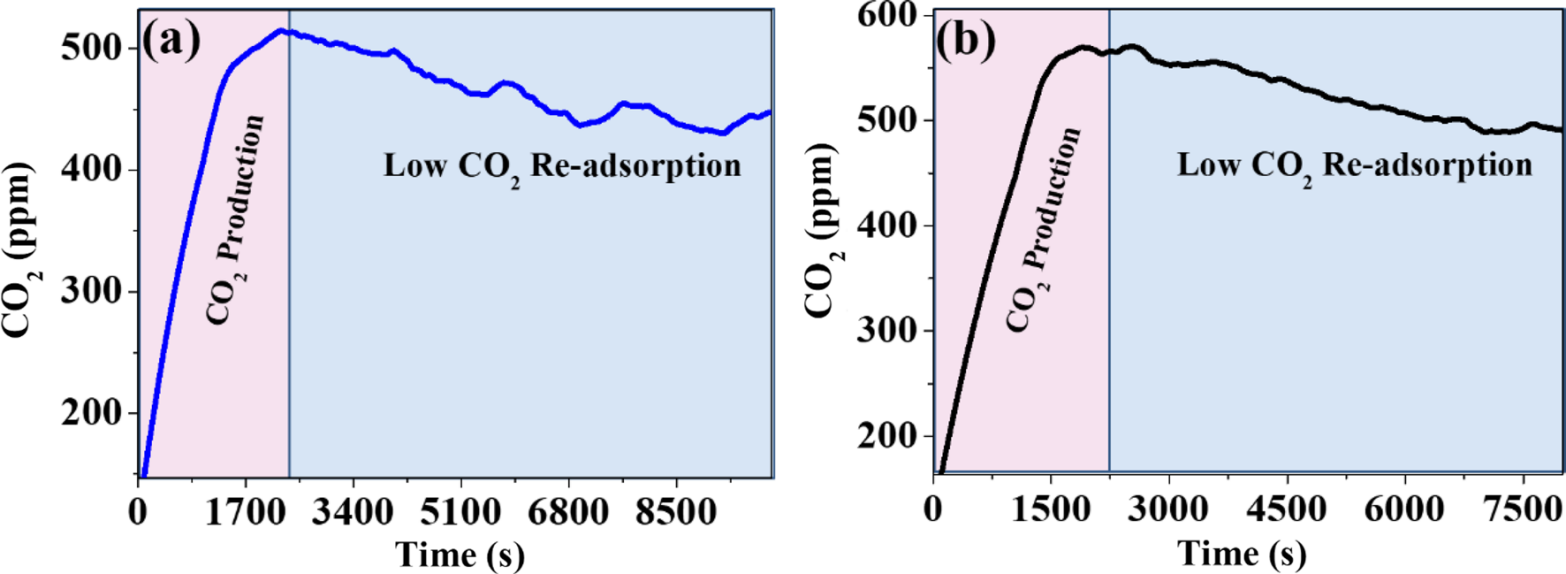
(**a**) GO 9.00% at 260.15 K and (**b)** GO 9.00% at 253.15 K.

The graphene’s were also exposed to low temperatures (ranging from 260.15 to 253.15 K) obtaining favorable low re-adsorption results, as seen in Fig. 8a, because of less than 10% re-adsorption. This figure shows how very low graphene oxide adsorbed more CO_2_ from the system. After being exposed to low temperatures for several hours, it intervened in the CO_2_ saturation obtained from previous experiments, starting from a concentration of 100 ppm before the CO_2_ production reaction and with a maximum CO_2_ concentration of 550 ppm, at 2000 s; after this time, a decrease in concentration of approximately 450 ppm is obtained in 8500 s. As in the previous experiment, it is shown how very low graphene oxide adsorbed more CO_2_ from the system after exposure to low temperatures of 253.15 K for 24 h, starting from a CO_2_ concentration of 100 ppm and a maximum of 550 ppm of carbon dioxide at 2000 s, but after this time a decrease in concentration of approximately 480 ppm was obtained in 7500 s; where low CO_2_ adsorption is observed, as identified in Fig. 8b. It would be very important to continue exploring even with lower temperatures, given that if temperature is too low, the CO_2_ molecules can freeze and become less mobile, which could decrease the total re-absorption amount, which serves as another desorption method.

Table 3 shows the different materials used in this work for CO_2_ capture. The three oxidation rates of graphene are included, the zeolite and the silica gel, listed with their respective efficiencies, yields, system temperatures, and humidity. The methods to estimate efficiency and yield are briefly described in the Supplementary information.

**Table 3:** Efficiency (η) v/s yield (Y), with their respective system *Room temperature (Rt), humidity (h) and atmospheric pressure (hPa).

Types of materials	η (efficiency) (%)	Y (yield) (%)	Rt* (K)	h (%)	Atmospheric pressure (hPa)
GO 9.00%	86.28	99.50	294.15	69	853.26
GO 5.25%	89.38	60.10	300.15	55	855.26
GO 3.00%	92.20	86.60	295.15	70	850.20
Zeolite	49.75	97.04	295.15	70	851.27
Silica gel	54.41	97.67	295.15	70	853.21

### Theoretical results

**Figure 9:**
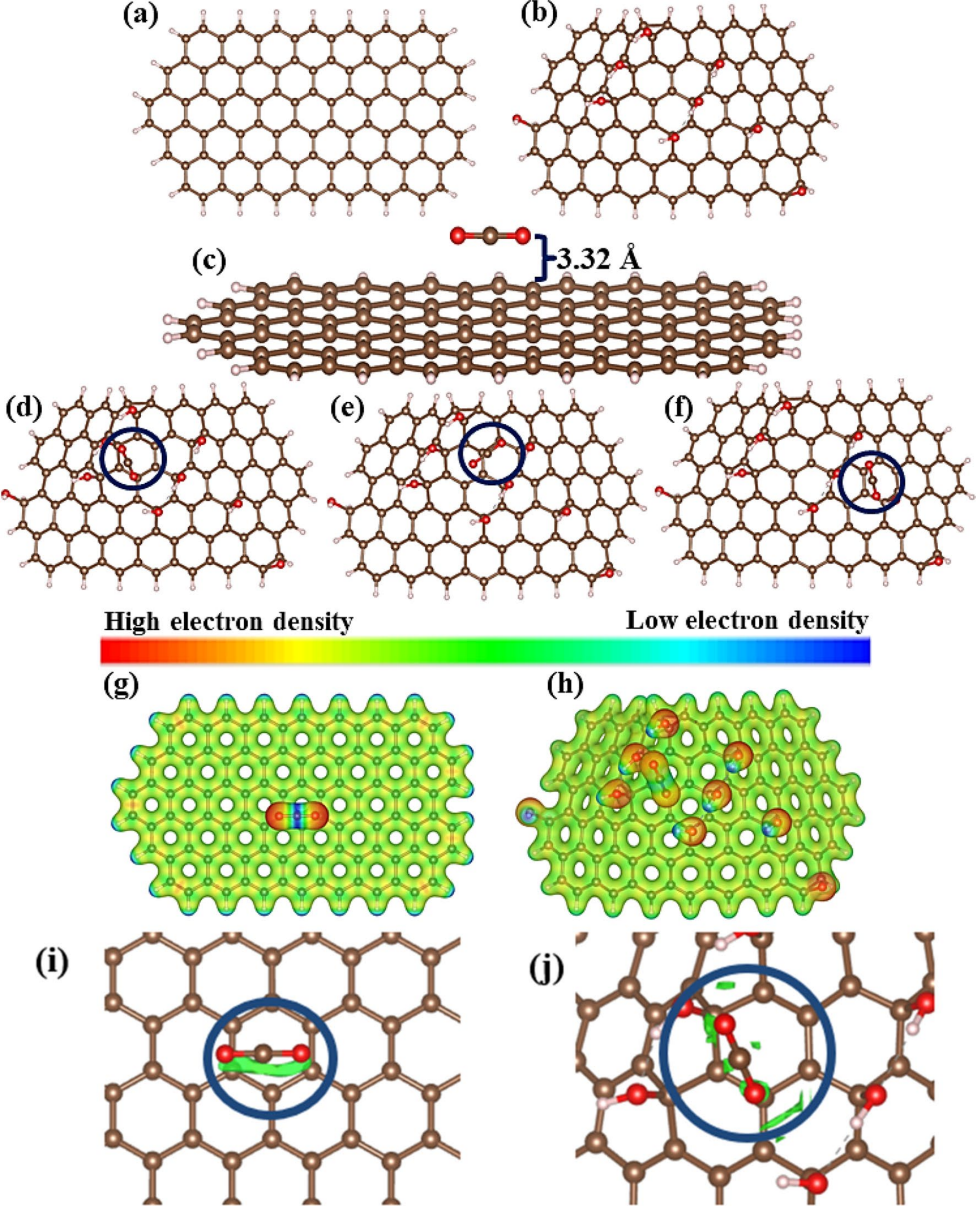
Structures studied. (**a**) Graphene, (**b**) GO with hydroxyl bridges, (**c**) CO_2_ molecule adsorbed in pristine graphene passivated by hydrogen atoms in its edges, (**d**) converged CO_2_/GO structures for the GO-1 position, (**e**) GO-2 position, (**f**) GO-3 position, (**g**) MEP for pristine graphene/CO_2_, (**h**) MEP for GO*-*1 position, (**i**) NCI for pristine graphene/CO_2_ and (**j**) NCI for GO-1 position.

The optimized structures can be seen in Fig. 9, showing the positions of the functional groups: hydroxyl (–OH) and epoxy (–O–). On the surface of the final relaxed graphene structure, the CO_2_ molecule was placed at 3.32 Å, as shown in Fig. 9c, consistent with that reported in the literature^84^, suggesting weak interactions, like Van Der Waals and NCl. The CO_2_ molecule was positioned in three locations, the first one, GO-1, between the bottom hydroxyl groups, the second one, GO-2, at the top hydroxyl of the structures and last, GO-3, near the single hydroxyl on the right of the structure, which correspond to Fig. 9d–f, respectively.

Figure 9g and h presents MEP calculation images and reveals a high or low electron density, presenting the reactivity point of the surface of GO structures. Comparing Fig. 9g and h, the charge redistribution is noted of the CO_2_ molecule due to the interaction with the GO structure in which the NCI displayed in Fig. 9i and j proves the existence of the weak relation between the two structures through the van der Waals interaction for each system.

The *E*_*ads*_ for each system is displayed in Table 4, which shows the adsorption energy for the CO_2_/Graphene system, (− 0.2288 eV), agreeing with the values reported by Wang et al.^85^ and the decreasing trend of the values from graphene to each position of CO_2_ in GO is visible and suggests physisorption, as the main adsorption mechanism; there is also the decreased distance between the contaminant molecule and the GO, associated with differences of the electrical dipoles^84^. The Bader charge of the GO-2 system (0.6349 *e*) compared to the other structures, exhibits the highest value and, therefore, it is feasible to assume a stronger interaction between the components, given a charge transfer from the oxygen atom to the hydrogen from the adjacent hydroxyl, making this group highly important for carbon capture, especially in the GO-2 position.

**Table 4:** Calculated properties and adsorption energy for the CO_2_/Graphene and CO_2_/GO systems.

System	${E}_{ads}$(eV)	D (Å)	Q (*e*)
CO_2_/Graphene	− 0.2288	3.3278	–
GO-1	− 0.2376	2.7699	0.6112
GO-2	− 0.2400	2.6415	0.6349
GO-3	− 0.2334	2.2500	0.6085

Calculated adsorption energy (${E}_{ads}$), distance from CO_2_ to the surface of graphene or hydroxyl for GO (D), charge transfer from the graphene and GO to CO_2_ (Q).

These results suggest a possible physisorption mechanism between the graphene and CO_2_, which describes Van Der Waals interaction between the GO and CO_2_, making these materials excellent candidates for carbon capture and air decontamination.

### Possible applications

**Figure 10:**
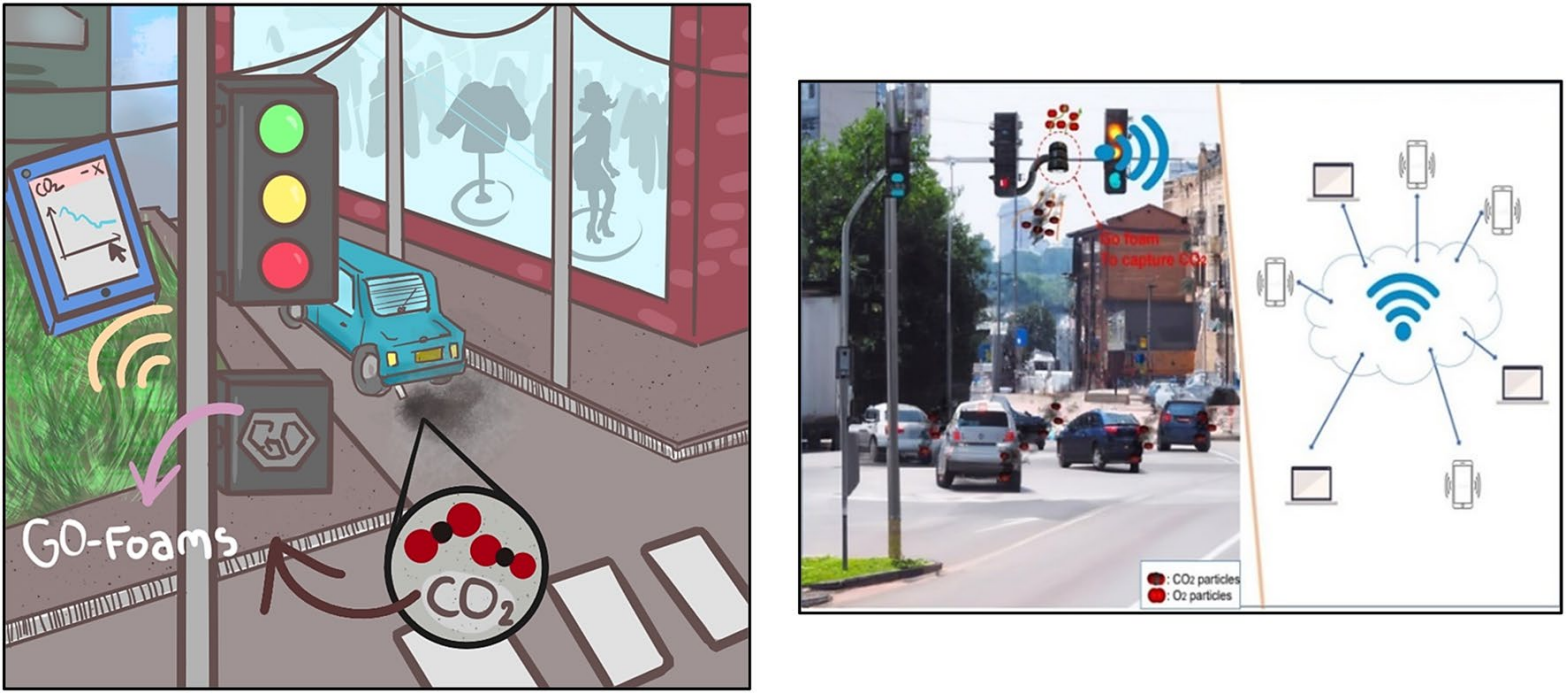
Possible application of GO–Foam–CO_2_ for carbon removal in a traffic light. (Permissions allowed by Erica Valencia (left figure) and Humberto Franco (right figure). copyright holders).

The GO–Foam–CO_2_ prototype could be used as a CO_2_ capture, purification, and monitoring system in many places, like parks, main squares, trains, planes, airports and, overall, in cities with high concentrations of CO_2_, as seen in Fig. 10.

## Conclusions

The results of heating the GO at 9%, 5%, and 3% to 673.15 K for several hours show successful results because GOs recover their conditions as adsorbent material. Conversely, when heating these GO below 673.15 K, it was observed that the GOs do not release the CO_2_ gas. It was noted how at specific low temperatures, in this case, 260.15 and 253.15 K, GOs do not recover their adsorption capacity; therefore, making a better sweep of this low-temperature area would be extremely important, for example where CO_2_ stops being gas. The organic materials used in this work to make an ideal comparison with non-adsorbent materials, in this case, roasted and dry coffee, are identified as non-adsorbent materials. Zeolite and silica gel in this work are used as a reference to a CO_2_ gas adsorbing material; therefore, it was quite comfortable to make the comparison with the three different oxidation rates of GO. The GO at 873.15 K had the best performance, but the GO at 1053.15 K had the highest efficiency. The relaxed structures present adsorption values in the weak physisorption range, indicating interactions of the hydroxyl groups on the surface of GO with the CO_2_ molecule, which can be interpreted that GO is a promising material for carbon capture from the air and opens the possibility of developing technological devices with these types of materials. It was also of great importance to find that this material can desorb at 673.15 K. These results suggest that GO foams are a promising material for carbon capture and future development of a new clean tech, given their highest CO_2_ adsorption efficiency and yield.

## Supplementary Information

Supplementary Information.

## Data Availability

The datasets used and/or analyzed during the current study are available from the corresponding author upon reasonable request.
