# Author Correction: Exploring the capture and desorption of CO_2_ on graphene oxide foams supported by computational calculations

**DOI:** 10.1038/s41598-025-88643-8

**Published:** 2025-02-12

**Authors:** Bryan E. Arango Hoyos, H. Franco Osorio, E. K. Valencia Gómez, J. Guerrero Sánchez, A. P. Del Canto Palominos, Felipe A. Larrain, J. J. Prías Barragán

**Affiliations:** 1 Energy Engineering, Faculty of Engineering and Sciences, Universidad Adolfo Ibáñez, 7941169 Santiago, Chile; 2 Electronic Instrumentation Technology Program, Faculty of Basic Science and Technology, Universidad del Quindío, 630001 Armenia, Colombia; 3 Doctoral Program in Physical Sciences, Interdisciplinary Institute of Sciences, Universidad del Quindío, 630004 Armenia, Colombia; 4 Virtual Materials Modeling Laboratory (LVMM), Center for Nanoscience and Nanotechnology, Universidad Nacional Autónoma de México, Ensenada, 22860 Mexico

Correction to: *Scientific Reports* 10.1038/s41598-023-41683-4, published online 02 September 2023

The original version of this Article contained errors in Table 1, where the “Average capacity (mmolCO2/g adsorbent)” value for “PEI/silica” was incorrect. The correct and incorrect values appear below.

Incorrect:

**Table:** 

Materials	Method	Sample	Cycle time (min)	Stability evaluation (cycles)	Average capacity (mmolCO_2_/g adsorbent)	Ref.
PEI/silica	TGA	Pelletized composite	465	4	7.50	^6^

Correct:

**Table:** 

Materials	Method	Sample	Cycle time (min)	Stability evaluation (cycles)	Average capacity (mmolCO_2_/g adsorbent)	Ref.
PEI/silica	TGA	Pelletized composite	465	4	2.26	^6^

The original Article has been corrected.

