# Supplementary material for: Exploring the capture and desorption of CO2 on graphene oxide foams supported by computational calculations
Source: Sci Rep. 2023 Sep 2;13:14476. doi: 10.1038/s41598-023-41683-4 (PMC10475065; doi:10.1038/s41598-023-41683-4)
Supplement: Supplementary file 1 — Supplementary Information. [file 41598_2023_41683_MOESM1_ESM.pdf]

Exploring the Capture and Desorption of CO_2 on Graphene Oxide Foams Supported by Computational Calculations

B. E. Arango Hoyos [1,5], H. Franco Osorio [2,5], E. K. Valencia Gómez [3,5], J. Guerrero Sánchez [4,5], A. P. Del Canto Palominos [1,5], F. A. Larraín Benavides [1,5] and J. J. Prías Barragán [2,3,5,*]

[1] Engineering Program, Faculty of Engineering and Sciences, Universidad Adolfo Ibáñez, Santiago, 7941169, Chile.
[2] Electronic Instrumentation Technology Program, Faculty of Basic Science and technology, Universidad del Quindío, Armenia, 630001, Colombia.
[3] Doctoral Program in Physical Sciences, Interdisciplinary Institute of Sciences, Universidad del Quindío, Armenia, 630004, Colombia.
[4] Virtual Materials Modeling Laboratory (LVMM), Center for Nanoscience and Nanotechnology, Universidad Nacional Autónoma de México, Ensenada, 22860, México.
[5] All authors contributed equally to this article.
* Corresponding author E-mail: jjprias@uniquindio.edu.co

Supplementary Information

Other organic materials that do not absorb CO_2

A. Dry coffee

The production of CO_2, was prepared at the lab with 1.5 mg of $NaHCO_{3(solid)}$ (sodium bicarbonate) plus 0.5 ml of $CH_3COOH_{(aqueous)}$ (acetic acid). In this experiment, "various formats of coffee" were used as non−absorbent material to be able to buy absorbent materials, specifically one called "E2 rich" in this dry case from a region of Colombia called Quindío. In Figure S1 using the sensor MHZ−19B, it can be seen how there is a CO_2 production with a maximum of 700 ppm, and as the experience elapses, how the CO_2 gas production remains constant show how not absorb.

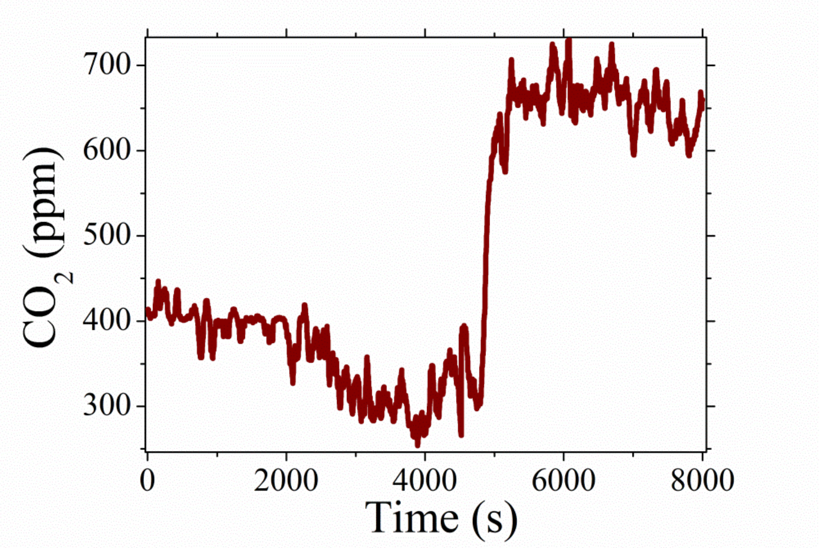

Figure S1 *Dry coffee material without CO_2 absorption.*

B. Toasted coffee

In this experiment (E23), for the production of CO_2, 1.5 mg of $NaHCO_{3(solid)}$ (sodium bicarbonate) plus 0.5 ml of $CH3COOH_{(aqueous)}$ (acetic acid) were used. In this experience, roasted coffee from Belén de Umbría, Risaralda, Colombia, is specifically used. In figure 6.1, using the sensor MQ−135, the CO_2 production always increases, approaching 3500 ppm and then forming constant valley of CO_2 production stability, showing that it is not a material that can absorb CO_2. In Figure S2 using the sensor MHZ−19B, there is a CO_2 production with a maximum of 1200 ppm, and as the experience elapses, the CO_2 gas production remains constant.

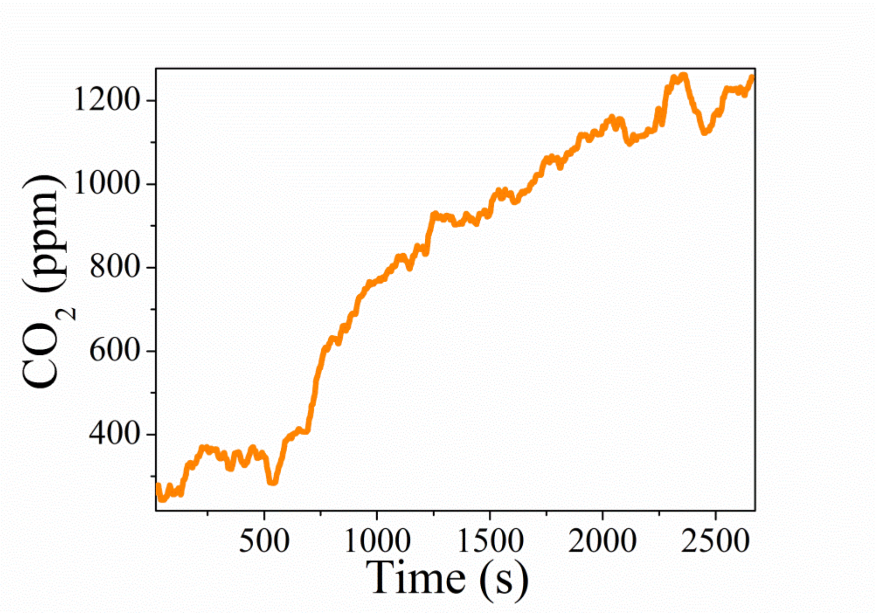

Figure S2 *Toasted coffee material without CO_2 absorption.*

Method for calculating efficiency (η) and yield (Y)

The efficiency and yield were calculated by means of the curves thrown by the measurement of the MHZ−19B sensor, where the data of the maximum CO_2 production was taken and then the data of the minimum CO_2 adsorption, for both the concentration and the elapsed time. For the efficiency the time in seconds were used and for the yield the elapsed the concentrations in (ppm) was used and all this multiplied by one hundred.
